# Integrated Low-Voltage Compliance and Wide-Dynamic Stimulator Design for Neural Implantable Devices

**DOI:** 10.3390/s23010492

**Published:** 2023-01-02

**Authors:** Yeonji Oh, Jonggi Hong, Jungsuk Kim

**Affiliations:** 1 Department of Medical Science, Korea University, Seoul 02841, Republic of Korea; 2 Department of Health Sciences & Technology, Gachon Advanced Institute for Health Sciences & Technology, Gachon University, Incheon 21999, Republic of Korea; 3 Department of Biomedical Engineering, Gachon University, Incheon 21936, Republic of Korea

**Keywords:** functional electrical stimulation, retina implant, neuromorphic system, pulse frequency modulation, subretinal, sim4life

## Abstract

In this study, a pulse frequency modulation (PFM)-based stimulator is proposed for use in biomedical implantable devices. Conventionally, functional electrical stimulation (FES) techniques have been used to reinforce damaged nerves, such as retina tissue and brain tissue, by injecting a certain amount of charge into tissues. Although several design methods are present for implementing FES devices, an FES stimulator for retinal implants is difficult to realize because of the chip area, which needs to be inserted in a fovea, sized 5 mm x 5 mm, and power limitations to prevent the heat generation that causes tissue damage. In this work, we propose a novel stimulation structure to reduce the compliance voltage during stimulation, which can result in high-speed and low-voltage operation. A new stimulator that is composed of a modified high-speed PFM, a 4-bit counter, a serializer, a digital controller, and a current driver is designed and verified using a DB HiTek standard 0.18 μm process. This proposed stimulator can generate a charge up to 130 nC, consumes an average power of 375 µW during a stimulation period, and occupies a total area of 700 µm × 68 µm.

## 1. Introduction

**Figure 1:**
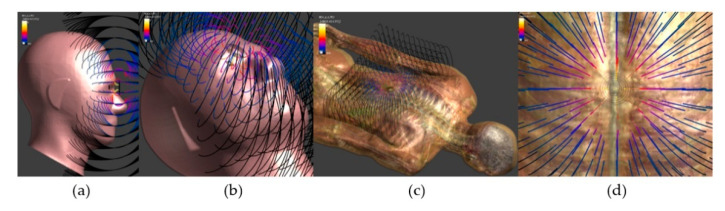
Examples of retinal prosthetics. (**a**) side view (**b**) whole view, spinal stimulation device (**c**) whole view (**d**) front view (simulated by Sim4life, human model software of IT’IS Foundation (Information Technologies in Society)).

Functional electrical stimulation (FES) techniques have been used to artificially restore impaired neuronal activity by injecting low-energy electrical pulses into the damaged site [1,2]. FES is used for spinal cord injuries [1,2], bladder control [3], and retinal prosthetics [4,5]. Figure 1 illustrates representative examples in which the spinal stimulation device and retinal prosthetics are applied to the human body. Among FES applications, many researchers have recently focused on retinal prostheses to overcome technical limitations.

Retinal prosthesis is a device used to regain vision in patients with retinal degenerative disease. Age-related macular degeneration (AMD) and retinitis pigmentosa (RP) are the two most prevalent retinal degenerative diseases [4,6]. Most of these patients lose photoreceptors, but retinal neurons in the inner null layer and ganglion cell layer remain alive with a high probability [5,7]. The retinal prosthesis electrically stimulates the remaining retinal neurons using a microelectrode array inserted between the degenerated photoreceptor layer and the retinal pigmented epithelium (RPE) [8]. Research on the artificial retina has been carried out previously; thus, some retinal prostheses have been clinically tested on the human body, such as BVA suprachoroidal stimulation devices with 44 electrodes [9], Argus II devices with 60 electrodes [10], and Alpha AMS with 1600 electrodes [11]. These studies show that stimulating the remaining retinal cells can evoke a phosphene or visual sensation.

Advantageously, the subretinal prosthesis can use existing neural processing in the outer and middle layers of the retina, possibly avoiding the direct stimulation of ganglion cell axons that distorts visual perception [12]. In addition, a subretinal prosthesis can provide high-resolution pixels on a limited chip area [13], which is determined by the size of the fovea.

For the subretinal implant, a photodiode is used to sense the light intensity. However, it is difficult to directly stimulate the remaining retinal cells using a dark current because the current is too small to excite the cell. Accordingly, it is necessary for the subretinal implant to use a current amplifier to amplify the minute dark current, and a pulse shaper to generate biphasic electrical pulses. To amplify the photodiode current, a capacitor is harnessed to accumulate the charge defined by the current multiplied by the integration time [14]. However, the amount of charge is not linearly proportional to the light intensity because of the mismatch of the switched capacitors used for the current mirrors. In addition, the previous photocurrent amplifier, which is composed of complex analog circuitry, occupies a large layout area, making it difficult to achieve high-density stimulation on a limited chip area. To overcome this disadvantage, various pulse-frequency modulation (PFM) techniques have been devised for hardware simplicity and high linearity [15]. In addition, PFM is more effective for cell stimulation because it generates a pulse stream output. Static stimulation rarely induces cell potentials [14]. Previous PFM circuits slowly digitize the analog dark current; therefore, it is difficult to apply the PFM structure to real-time image processing in a high-resolution subretinal implant. Therefore, it is essential to increase the digitization speed of the PFMs. 

**Figure 2:**
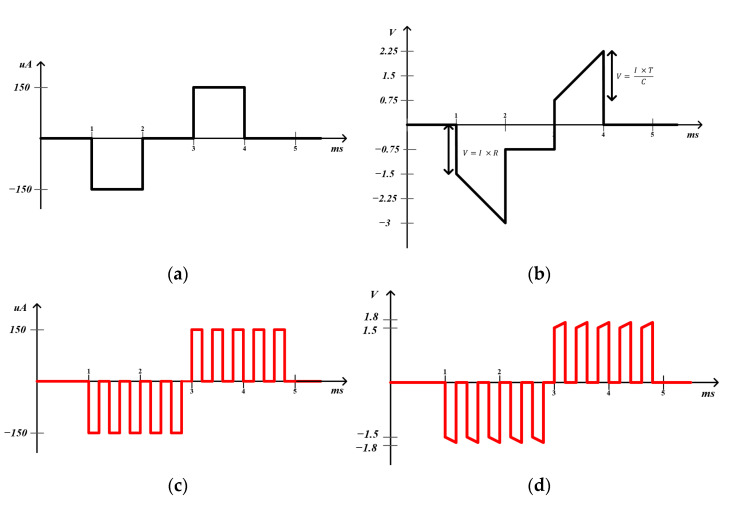
Examples of current–time and voltage–time graphs to compare conventional biphasic stimulation with proposed biphasic stimulation. (**a**) Current–time graph of single biphasic pulse for 150 nC (*i* = 150 μA, *t* = 1 ms) (**b**) Voltage–time graph of single biphasic pulse. Depending on the typical electrode model value [16], C is set at 100 nF and R is set at 10 KΩ. Furthermore, 1.5 V voltage is defined by Ohm’s law (V = I × R). Compliance voltage is 1.5 V, which can be obtained from equation Q=i×t=C×V. V  can be defined as  i×t / C. (**c**) Current–time graph of proposed biphasic pulse for 150 nC (five pulses with I = 150 μA, *t* = 200 μs) (**d**) Voltage–time graph with a reduced compliance voltage using proposed biphasic pulses. Compliance voltage is 0.3 V, which is a result of decrease in the charge injection duration time.

To accomplish both high-resolution stimulation array and high-speed image processing, the PFM circuit should consider the following three challenging design issues. First, the photosensor must have the ability to promptly digitize the change in dark current. As the dark current is too small to be directly sensed by a digitizer, it is necessary to increase the digitization speed of the PFM-based stimulator for real-time image processing on the retinal chip. Second, the simulator output must have a wide dynamic range. Figure 1a shows a current–time graph of the traditional biphasic stimulation method, and Figure 2b is a voltage–time graph considering the electrode. Voltage increases due to the capacitor in the tissue–electrode interface model [16]. The rising compliance voltage makes MOSFET of output shrink, which can shift MOSFET from the saturation region to the triode region. These changes can be a factor in poor MOSFET performance, so we propose the PFM-based stimulation scheme, as shown in Figure 2c,d. To avoid high-compliance voltage, we decrease the charge injection duration time. Through this strategy, the stimulus output has lower-compliance voltage with the same amount of current and has a wider output dynamic range. Finally, the active area of the stimulator must be as small as possible to increase pixel density. Previous studies have required an integration capacitor to accumulate a charge and a current-steering digital-to-analog converter (DAC) to control the amplitude of the biphasic current, which results in a large active area [17]. To the best of our knowledge, a subretinal stimulator that meets all of the above requirements has not yet been developed. Motivated by this, in this paper, we propose a novel PFM-based stimulator that simultaneously achieves high-speed, high-resolution, and low-compliance output voltage by creating a biphasic pulse train using a digital logic block, a 4-bit counter, and a serializer without using a conventional DAC. The proposed stimulator circuit was designed and verified using a standard 0.18 µm 1P6M CMOS process. 

## 2. Methods

### 2.1. System Architecture

**Figure 3:**
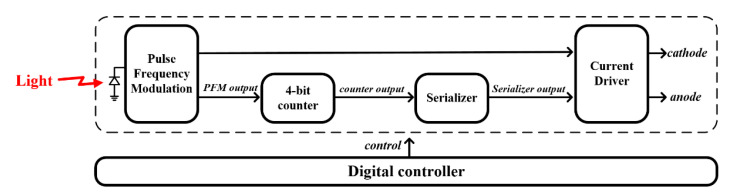
A proposed one-pixel stimulator architecture composed of a pulse-frequency modulation, a 4-bit counter, a serializer, and a current driver. By rejecting a conventional DAC block, we can reduce the proposed system’s active area in this design.

**Figure 4:**
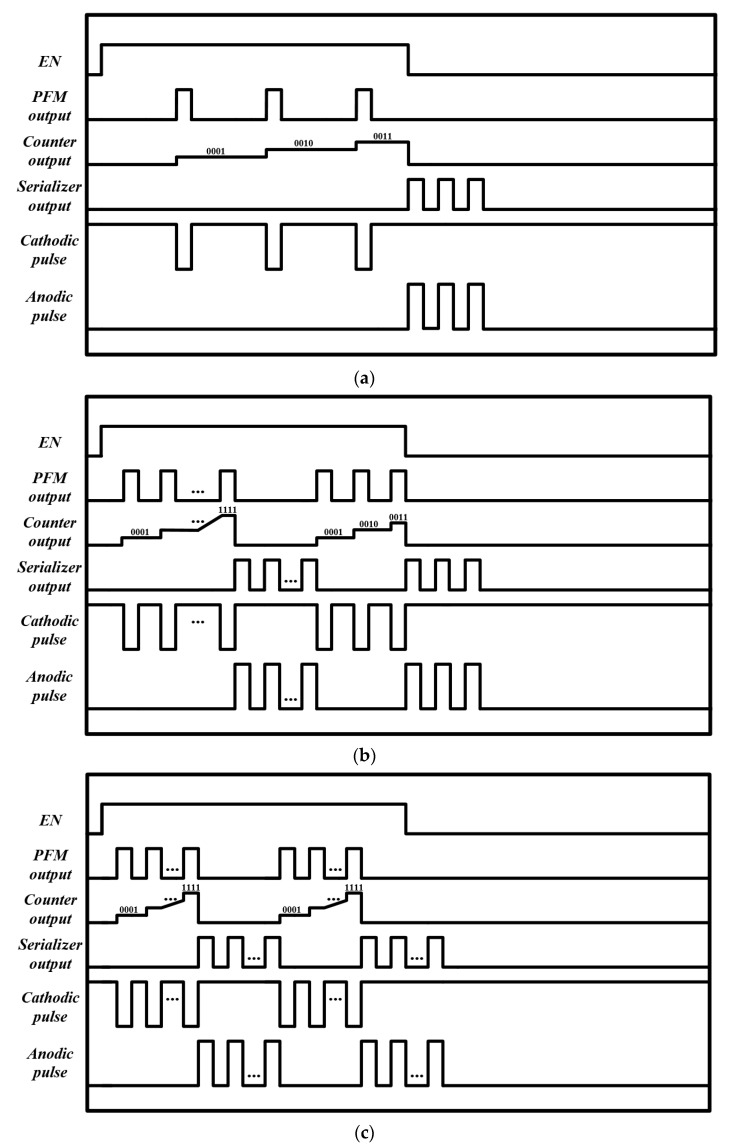
Timing diagram example of the proposed system. (**a**) The incoming light generates only three pulses. (**b**,**c**) The incoming light generates pulse that is composed of more than 16 pulses during enable high.

Figure 3 depicts the proposed one-pixel stimulator system architecture, which consists of five functional blocks: pulse frequency modulation (PFM), 4-bit counter, serializer, current driver, and digital controller. The PFM senses incoming light using a photodiode during an enable time, as shown in Figure 4, and outputs digitized pulses, the number of which depends on the light intensity. In this study, pulse trains were employed to generate cathodic pulses through the current driver. Meanwhile, the 4-bit counter started counting the number of PFM output pulses, which were used to produce anodic pulses after cathodic pulses. 

Figure 4 illustrates three timing-diagram examples to address each block’s functionality. An able signal selects a pixel by being high for a certain period. During the enable high, the photodiode in the PFM can sense light intensity. The PFM output was sent directly to the current driver to produce cathodic pulses and to the 4-bit counter to count the number of PFM output pulses. In this work, we set the enable time length to 5 ms, and the high lasts for 2.5 ms while the low maintains for 2.5 ms. The operation of the enable period is divided into three cases, as follows. 

In the first case, the photodiode in the PFM senses weak light intensity. As a result, the PFM output generates fewer than 16 pulse trains during the enabled high time. Figure 4a shows an example in which the PFM produces only three pulses during the enabled time. Each pulse enters the 4-bit counter, which can count a maximum of 16 numbers and also memorizes the number of pulses. Meanwhile, PFM pulses that directly move to the current driver are transformed into cathodic pulses. When the enable decreases, the serializer block starts generating flag signals corresponding to the number of PFM output pulses, which are transformed into anodic pulses passing through the current driver. The total number of cathodic pulses was equal to the number of anodic pulses during one stimulation cycle. This made it possible to reduce the residual charge on the tissue after stimulation. 

Figure 4b,c show the cases wherein the light intensity is strong enough, resulting in several stimuli sets composed of more than 16 pulses during the enable high. When the PFM output pulses reach 16 within the enable high, the digital controller generates a disabled signal to turn off the PFM operation. Meanwhile, the serializer serializes the parallel data generated by the 4-bit counter to output anodic pulse trains. When the number of anodic pulses was the same as the number of cathodic pulses, the digital controller activated the signal to turn on the PFM again. If the number of PFM pulse trains is not a multiple of 16 when the enable is low, as shown in Figure 4b, the system works like the case of weak light intensity mentioned above.

Figure 4c shows that, when the number of PFM pulse trains is multiple (16), the enable becomes low. In this case, regardless of whether it is high or low, the serializer serializes the parallel data generated by the 4-bit counter. A detailed description of each circuit is provided below:

### 2.2. Circuit Implementation

#### 2.2.1. Pulse-Frequency Modulation Circuit 

**Figure 5:**
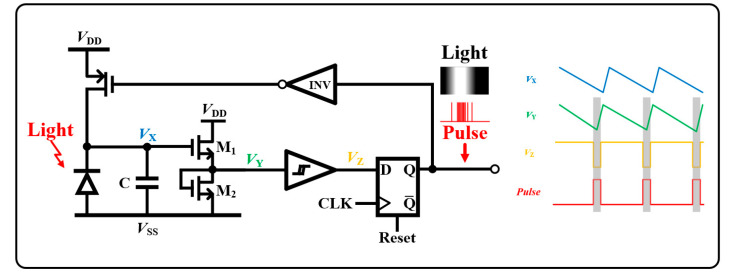
Proposed pulse frequency modulation circuit diagram where a source follower is newly added to digitize a dark current in high speed.

Figure 5 depicts the proposed PFM circuit, which digitizes the dark current produced by the photodiode. When light enters the photodiode, the voltage node of *V*_X_ drops because the parasitic capacitor *C*_P_ is discharged by the dark current. In previous studies, the node of *V*_X_ was directly connected to a Schmitt trigger [18] or an inverter [19] for digitization. However, the discharging speed is not sufficient to drive the Schmitt trigger or comparator, thereby generating slow-speed pulse trains at the PFM output. This is insufficient to promptly process the captured images for high-resolution retinal applications. To overcome this technical limitation, in this study, we devised a high-speed PFM structure by adding a source follower, followed by the photodiode illustrated in Figure 5. 

The source follower works to increase the voltage-drop speed at *V*_X_ using Equation (1), which has a non-inverting gain over 1.

(1) $$Av=\frac{Resistance\;tied\;between\;Source\;and\;GND}{\frac{1}{g_{m1}}+Resistance\;tied\;between\;Source\;and\;GND}=1+\frac{g_{m1}}{g_{m2}}$$

Owing to the input–output characteristic of the source follower, the voltage drop of *V*_Y_ becomes faster than that of *V*_X_. This resulted in high-speed output pulses. To digitize the source-follower output, there are three options: (1) a compactor, (2) an analog-to-digital converter, and (3) a Schmitt trigger. However, to reduce power consumption and achieve architectural simplicity, we chose a Schmitt trigger in this design. Herein, D flip-flop is employed to synchronize the digitized signal by the Schmitt trigger with a clock that is recovered by the wireless power carrier signal. The D flip-flop reset, which is manipulated by the digital controller, makes it possible to turn the PFM block on or off. This function assists in generating the anodic pulses described in the Section 2.1 (see Figure 4). 

In this work, the proposed PFM increased the linearity between the number of pulses and the light intensity projected onto the photosensor. 

#### 2.2.2. 4-Bit Counter and Serializer Circuit

**Figure 6:**
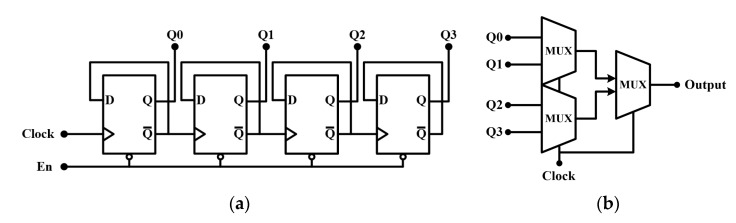
(**a**) 4-bit counter circuit that consists of four D flip-flops (**b**) serializer circuit composed of digital multiplexers.

Figure 6a,b shows the 4-bit counter and serializer structures, respectively. The 4-bit counter consists of four D flip-flops that can count and memorize the number of PFM output pulses. The results of *Q*_0_, *Q*_1_, *Q*_2_, and *Q*_3_ are sent to the serializer to generate anodic pulses. The serializer is made of three digital multiplexers with two inputs each, enabling four in one serializer. By implementing the counter and serializer with digital logic, we can achieve a low-area footprint on a chip.

#### 2.2.3. Current Driver Circuit 

**Figure 7:**
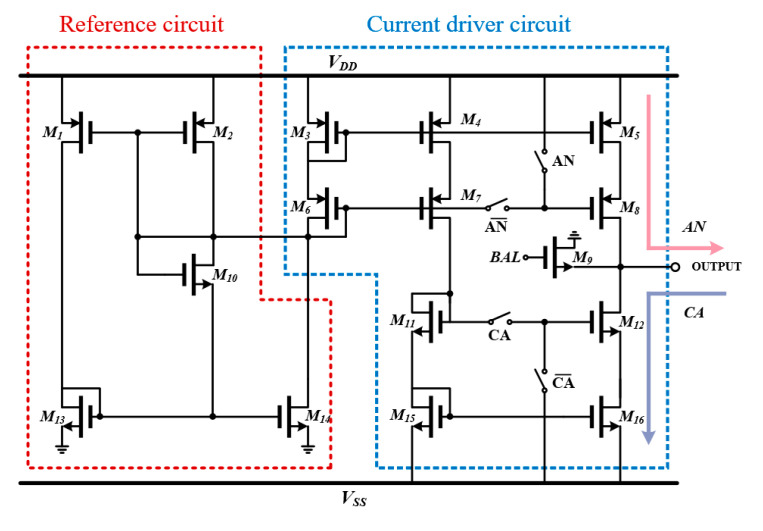
Push–pull structure-based biphasic current driver circuit wherein the reference current is supplied from a beta-multiplier reference circuit.

The current driver is composed of a current reference circuit and a push–pull structure controlled by the switches, AN and CA, to generate anodic and cathodic pulses as shown in Figure 7. We adopted a beta-multiplier reference for an independent reference rather than a bandgap reference because our human body maintains a constant body temperature of 37 °C. This reference circuit provides a current reference of 200 µA, a line regulation of 1.170 mV/V, and a power-supply rejection ratio of 54.92 dB. 

The cathodic switches of the current driver are directly connected to the PFM output, as illustrated in Figure 3, while the anodic switches are linked to the serializer output. To reduce the mismatch from the current mirrors, M5, M8, M12, and M13, we use a cascode structure for the output stage. In addition, to minimize charge residue after stimulation, a charge-balancing circuit composed of a single NMOS transistor with a high aspect ratio of 10 was harnessed in this design. With an enable time of 2.5 ms, when we set the system period of the clock to 8 µs, the current driver can generate 320 cathodic and anodic pulses during the enable high. This means that the total charge amount of one anodic (or cathodic) pulse is approximately 130 nC in one enable time for strong intensity light through Equation (2) (*i* = 200 µA, *t* = 4 µs, *N* = 160).

(2) $$Q_{T}\left(total\;charge\right)=i\times t\times N$$

## 3. Results

**Figure 8:**
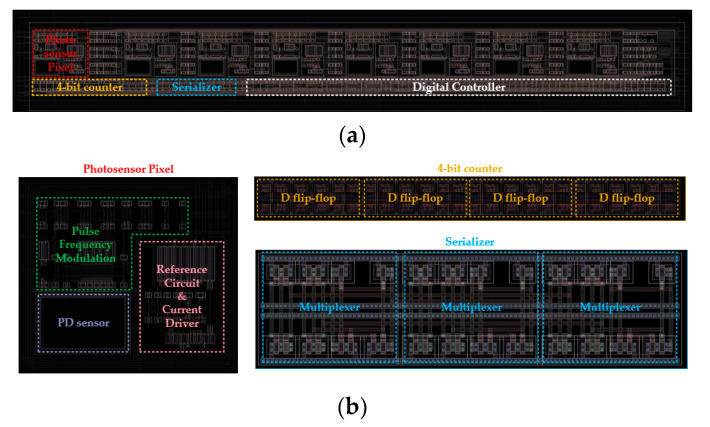
A footprint of the proposed 8-pixel circuit. (**a**) Full system with 8-pixel circuit that occupies 700 µm × 68 µm. (**b**) Footprint of each block that makes up the entire system. Single photosensor pixel area is 65 µm × 55 µm; 4-bit counter area is 145 µm × 10 µm. Serializer area is 45 µm × 10 µm.

Figure 8 shows the footprint of the proposed eight-pixel stimulation circuit that was designed using the DB HiTek 0.18 µm 1P6M CMOS process. As described in Section 2, we integrated eight stimulation pixels composed of a photodiode, a pulse frequency modulator, a 4-bit counter, a serializer, a current reference circuit, a current driver, and a digital controller onto a single silicon chip, which occupies a total active area of 700 µm × 68 µm. Accordingly, a single pixel had an area of 65 µm × 55 µm and had a power consumption of 375 µW. In the case of 2000 pixels, the total chip area is expected to be 3 mm × 3 mm, which will be fitted into a foveal area of 5 mm × 5 mm. 

**Figure 9:**
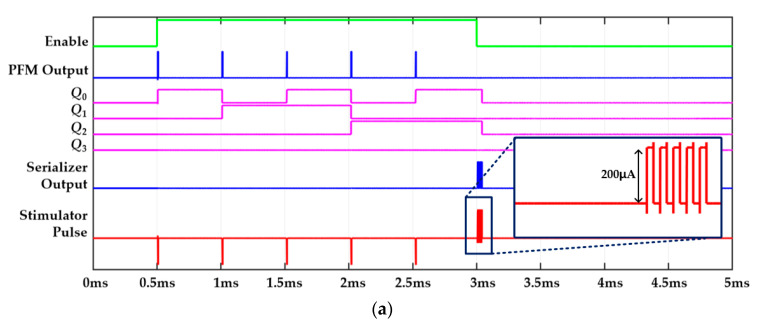

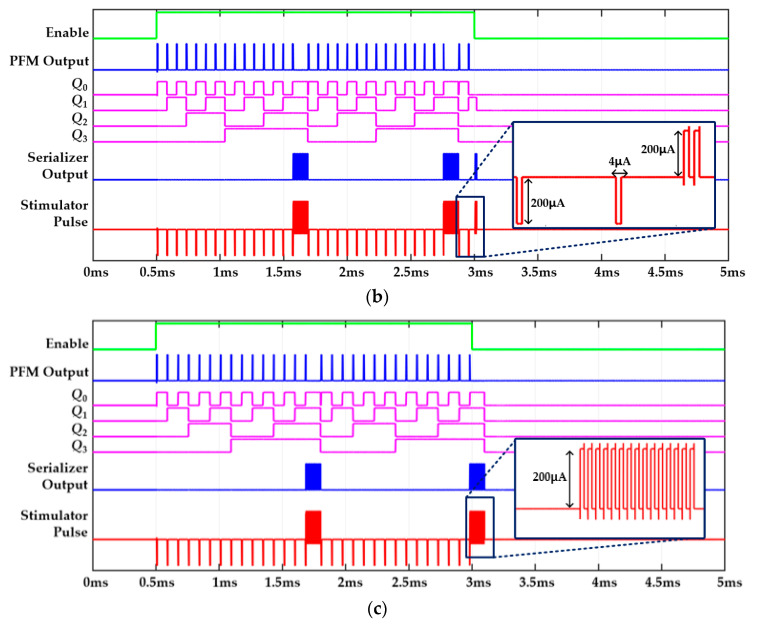
Observed transient output waveforms at each block. (**a**) Five pulses when the incoming light intensity is weak. (**b**,**c**) Two different cases when incoming light is strong (**c**).

Figure 9 shows the observed results of the proposed PFM-based high-speed stimulation pixel according to various light intensities. Figure 9a presents the case when the incoming light intensity is weak. Thus, the number of PFM output is lower than 16 pulse trains during the enable high time. The PFM output pulses are directly connected with the current driver, and as a result, the cathodic pulses are immediately generated as shown in the bottom red graph of Figure 9a. Meanwhile, the 4-bit counter also starts counting the number of PFM pulses that are depicted in *Q*_0_, *Q*_1_, *Q*_2_, and *Q*_3_ in the figure. Then, when the enable time becomes low, the counted results output anodic pulses passing through the serializer. The number of anodic pulses is exactly the same as the number of cathodic ones. This result shows that the proposed system can successfully cancel a residual charge after stimulation. 

Figure 9b,c illustrates the cases wherein the incoming light intensity is so strong that the PFM generates more than 16 pulse trains at the output. Figure 9b shows the case wherein the number of PFM pulses exceeds 16 pulses but not a multiple of 16 pulses within the enable high time of 2.5 ms. During the enable high time, regardless of when the 4-bit counter output reaches 16 (marked as ‘1111’ in the figure), the serializer and current driver activate to promptly generate anodic pulse trains (bottom red graph of Figure 9b). As soon as the enable becomes low, however, the counter stops working, and the serializer and current driver simultaneously start outputting cathodic pulses (see the black box of Figure 9b). Figure 9c shows the case in which the number of PFM pulses is multiples of 16. Two stimulation cycles were completed during the enabled high time. These results agree with the theoretically expected output (see Section 2.1). 

## 4. Conclusions

A low-compliance output voltage, low-area, and high-speed stimulator for artificial retinal implants was proposed and designed in this study. Conventional retinal implants have three main design challenges: (1) high-compliance output voltage during stimulation always requires high operating voltage, thereby resulting in high power consumption for the retinal devices; (2) it has been reported that real-time image processing is required to increase visual acuity for patients in whom the retinal prosthesis is implanted; (3) the stimulation pixel size should be as small as possible because of the limited area inside the eyeball. To meet these requirements, we propose a new PFM-based stimulator that consists of a 4-bit counter, serializer, and current driver. The modified PFM circuit makes it possible to digitize the dark-current variation promptly. In addition, the 4-bit counter and serializer, both of which are composed of digital logic, enable a small footprint and a low-compliance output voltage. The proposed PFM-based stimulator was designed and realized using a DB-HiTek 0.18 μm 1P6M process. The results showed that our stimulator achieved low-compliance voltage and high-speed stimuli in an active pixel area of 700 µm × 68 µm. In future work, we will design a neuromorphic image processor that can work with the proposed PFM-based stimulator and integrate more than 1600 pixels onto a single chip.

## Data Availability

The data presented in this study have been included in this article.
